# Type II-Activated Murine Macrophages Produce IL-4

**DOI:** 10.1371/journal.pone.0046989

**Published:** 2012-10-05

**Authors:** Anne Camille La Flamme, Marie Kharkrang, Sarrabeth Stone, Sara Mirmoeini, Delgertsetseg Chuluundorj, Ryan Kyle

**Affiliations:** 1 School of Biological Sciences, Victoria University of Wellington, Wellington, New Zealand; 2 Malaghan Institute of Medical Research, Wellington, New Zealand; Charité-University Medicine Berlin, Germany

## Abstract

**Background:**

Type II activation of macrophages is known to support Th2 responses development; however, the role of Th2 cytokines (esp. IL-4) on type II activation is unknown. To assess whether the central Th2 cytokine IL-4 can alter type II activation of macrophages, we compared the ability of bone marrow-derived macrophages from wild type (WT) and IL-4Rα-deficient mice to be classically or type II-activated *in vitro*.

**Results:**

We found that although both WT and IL-4Rα-deficient macrophages could be classically activated by LPS or type II activated by immune complexes plus LPS, IL-4Rα-deficient macrophages consistently produced much higher levels of IL-12p40 and IL-10 than WT macrophages. Additionally, we discovered that type II macrophages from both strains were capable of producing IL-4; however, this IL-4 was not responsible for the reduced IL-12p40 and IL-10 levels produced by WT mice. Instead, we found that derivation culture conditions (GM-CSF plus IL-3 versus M-CSF) could explain the different responses of BALB/c and IL-4Rα−/− macrophages, and these cytokines shaped the ensuing macrophage such that GM-CSF plus IL-3 promoted more IL-12 and IL-4 while M-CSF led to higher IL-10 production. Finally, we found that enhanced IL-4 production is characteristic of the type II activation state as other type II-activating products showed similar results.

**Conclusions:**

Taken together, these results implicate type II activated macrophages as an important innate immune source of IL-4 that may play an important role in shaping adaptive immune responses.

## Introduction

Macrophages can become activated by a variety of different products and signals [1]. One of the most commonly used macrophage activating products is lipopolysaccharide (LPS), the major outer membrane component of gram negative bacteria, and this microbial product classically activates macrophages into producing high levels of pro-inflammatory mediators such as IL-12, TNF-α, and IL-6 [2] and enhance the expression of CD40, PD-L1, CD86, and iNOS [3], [4]. This phenotype can promote the differentiation of naïve CD4 T cells into Th1 cells and has been shown to be protective during microbial infections and detrimental during specific inflammatory disease models such as EAE [1]. In contrast to classical activation, macrophages that are exposed to immune complexes (i.e. opsonized antigen; IC) in a pro-inflammatory environment (such as in the presence of LPS) become type II-activated [5]. These macrophages produce less IL-12 and significantly more IL-10 than classical macrophages while producing similar levels of TNF-α and NO [6]. Although the expression of CD40 and PD-L1 are significantly reduced, type II-activated macrophages are effective antigen-presenting cells and promote the development of Th2 cells both *in vitro* and *in vivo* [3], [7], [8]. This activation state can impair anti-microbial effector functions but has been shown to protect against pathology in pro-inflammatory disease models such as EAE and septic shock. Our previous work has shown that this activation state is protective during EAE, and this protection is dependent upon IL-4 [8]. However, it is unknown how the Th2 environment, promoted by type II-activated macrophages, is induced nor how this environment, once induced, affects the macrophage’s effector functions. To this end, we investigated if and how signaling through the major Th2 cytokine receptor IL-4Rα affected type II macrophage activation.

## Results and Discussion

### Bone Marrow-derived Macrophages Deficient in IL-4Rα Show Enhanced Classical and Type II Activation Compared to WT Macrophages

**Figure 1:**
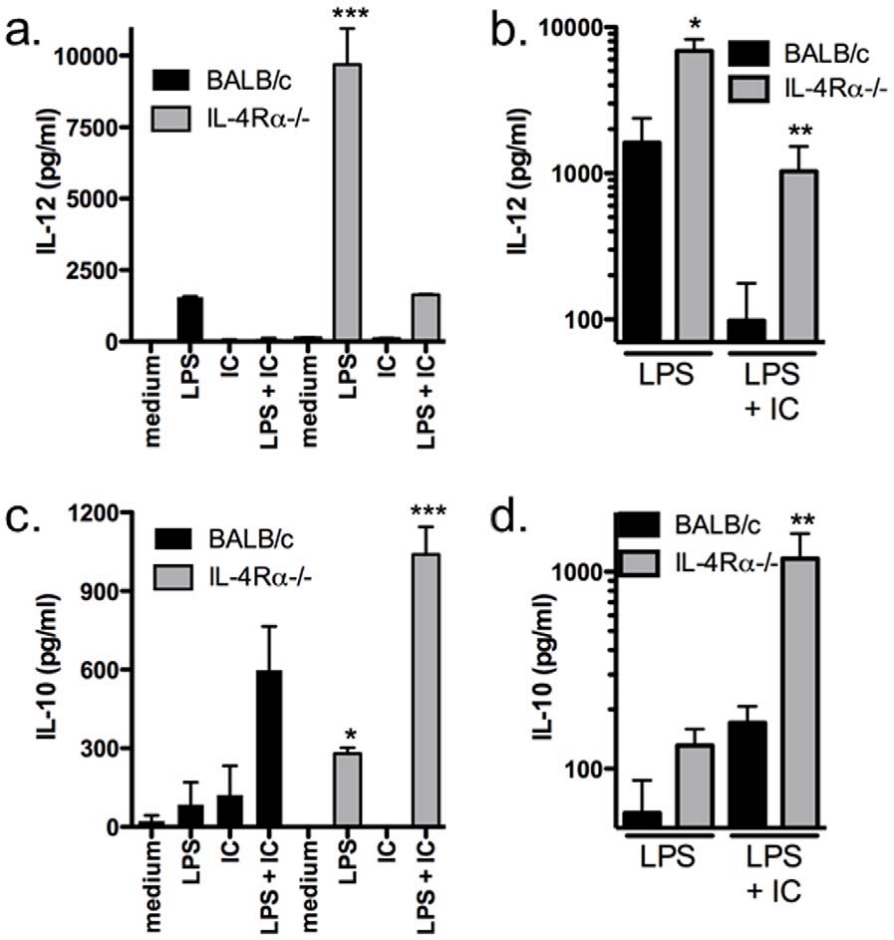
Type II-activated BMMφ from IL-4Rα-deficient mice produce significantly more IL-12p40 and IL-10 than WT BALB/c BMMφ. IFN-γ-primed BMMφ (10^5^/well) from WT and IL-4Rα-deficient mice were stimulated with LPS (10 ng/ml) in the presence or absence of opsonized SRBC (IC) for 24 hours. Cytokine production was measured in culture supernatants by ELISA. Shown are the means and SEM of triplicate wells from 1 of 3 experiments (a and c) or combined results from 4 experiments (b and d). ***p<0.001 compared to all other groups. **p<0.01 (b & d) LPS versus LPS + IC and (d) BALB/c versus IL-4Rα^−/−^. *p<0.05 (c) LPS versus medium or IC or (b) BALB/c versus IL-4Rα^−/−^. All p values were calculated by one-way ANOVA with Bonferroni’s post-test.

When IFN-γ-primed macrophages are stimulated by the TLR4 agonist LPS, they become classically activated and produce high levels of IL-12 [3]. In contrast, if FcγRs are ligated by immune complexes (IC) in addition to TLR4 activation, macrophages are type II-activated and preferentially produce IL-10 [3]. Because type II-activated macrophages have been shown to promote Th2 responses *in vitro* and *in vivo*, we investigated the role of the key Th2 cytokine, IL-4, in classical and type II activation. Using BMMφ from wild type (WT) and IL-4Rα−/− BALB/c mice, we compared the requirement for IL-4Rα signaling in activation. We found that both WT and IL-4Rα−/− BMMφ were able to become classically or type II-activated as shown by the IL-12 and IL-10 cytokine profiles (Figure 1). However, BMMφ deficient in IL-4Rα were found to produce significantly more IL-12 and IL-10 suggesting that signals through the IL-4Rα are involved in regulating the cytokine production of both classical and type II-activated macrophages.

### IL-4 is Produced by Type II Macrophages

**Figure 2:**
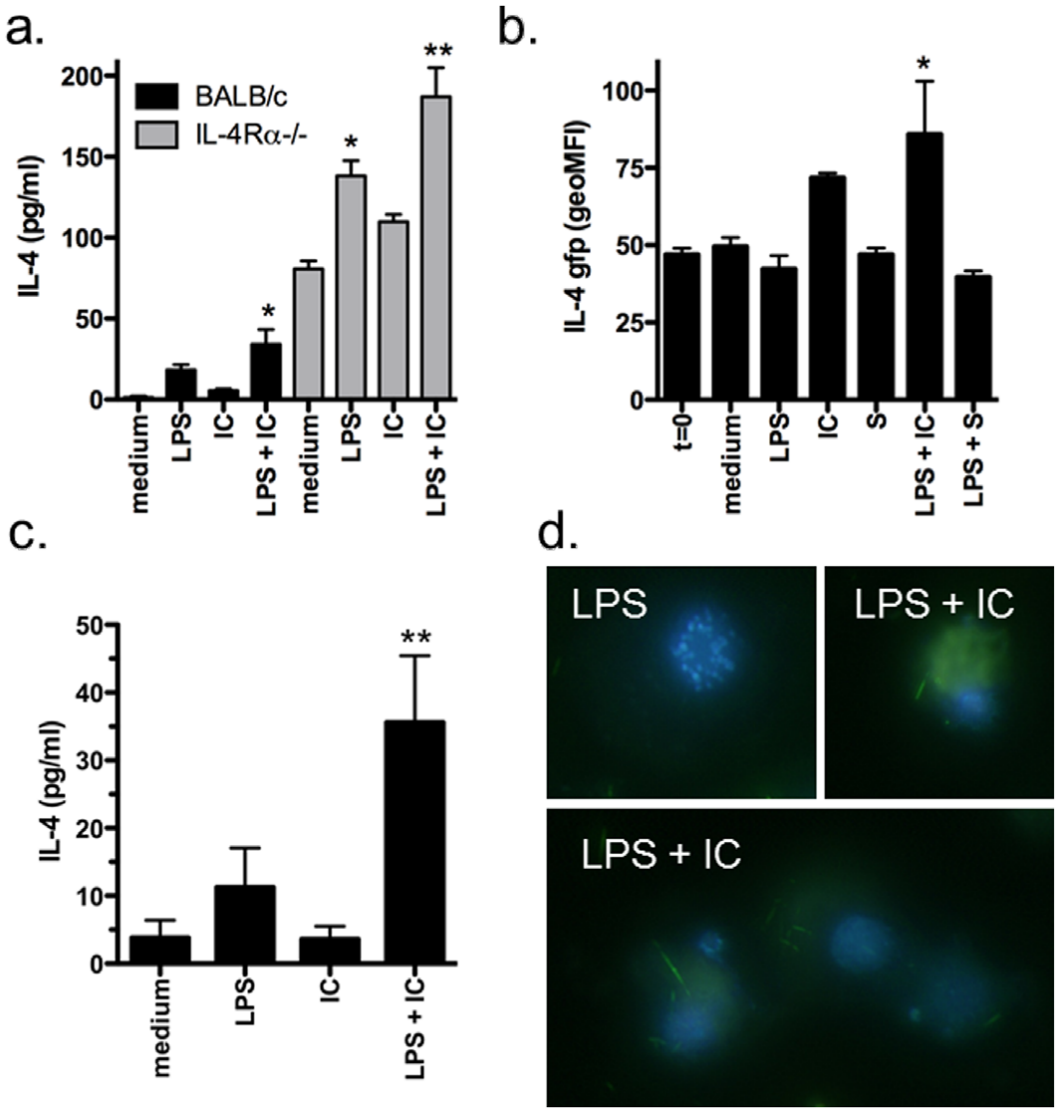
Type II-activated macrophages produce IL-4. (**a**) IFN-γ-primed BMMφ (10^5^/well) from WT and IL-4Rα-deficient mice were stimulated with LPS (10 ng/ml) in the presence or absence of opsonized SRBC (IC) for 24 hours. Cytokine production was measured in culture supernatants by ELISA. Shown are the means and SEM of triplicate wells from 1 of 3 experiments. *p<0.05 compared to medium and **p<0.001 compared to medium or IC. (**b–d**) IFN-γ-primed BMMφ (10^5^/well) from heterozygous G4 mice were stimulated with LPS (10 ng/ml) in the presence or absence of opsonized SRBC (IC) for 24 hours. IL-4 production by F4/80+ macrophages was assessed by flow cytometry (b). ELISA (c), or by fluorescence microscopy (d). IL-4-GFP is found intracellularly after LPS + IC (2 examples shown) but not LPS stimulation in DAPI-counterstained macrophages. Shown are the means and SEM from 3 experiments (b) or representative data from 1 of 3 experiments (c and d). *p<0.05 compared to t = 0 and **p<0.01 compared to medium by Dunnett’s Multiple Comparison Test.

A previous study reported the production of IL-4 by LPS-stimulated macrophages and found that at later time points (i.e. 24–48 hours) significant IL-4 was detected in the culture supernatants of stimulated macrophages [9]. Additionally IL-13, which also signals through IL-4Rα, has been reported to be produced by alveolar macrophages under specific conditions [10]. Thus to determine if IL-4 or IL-13 were produced after type II activation, we assessed IL-4 and IL-13 production by BMMφ from WT and IL-4Rα-deficient mice. In agreement with previous work, we found low levels of IL-4 were detected after stimulation of WT BMMφ with LPS, and this level increased upon type II activation (Figure 2a; black bars) and was detectable as early as 8 hours post stimulation (Figure S1). BMMφ from IL-4Rα-deficient animals produced far greater amounts of IL-4 than WT BMMφ (Figure 2a; grey bars), and this increase may reflect the build up of cytokine in the supernatant due to the absence of receptor uptake. In contrast to IL-4, we did not detect any increased production of IL-13 after classical or type II activation (Figure S2).

**Figure 3:**
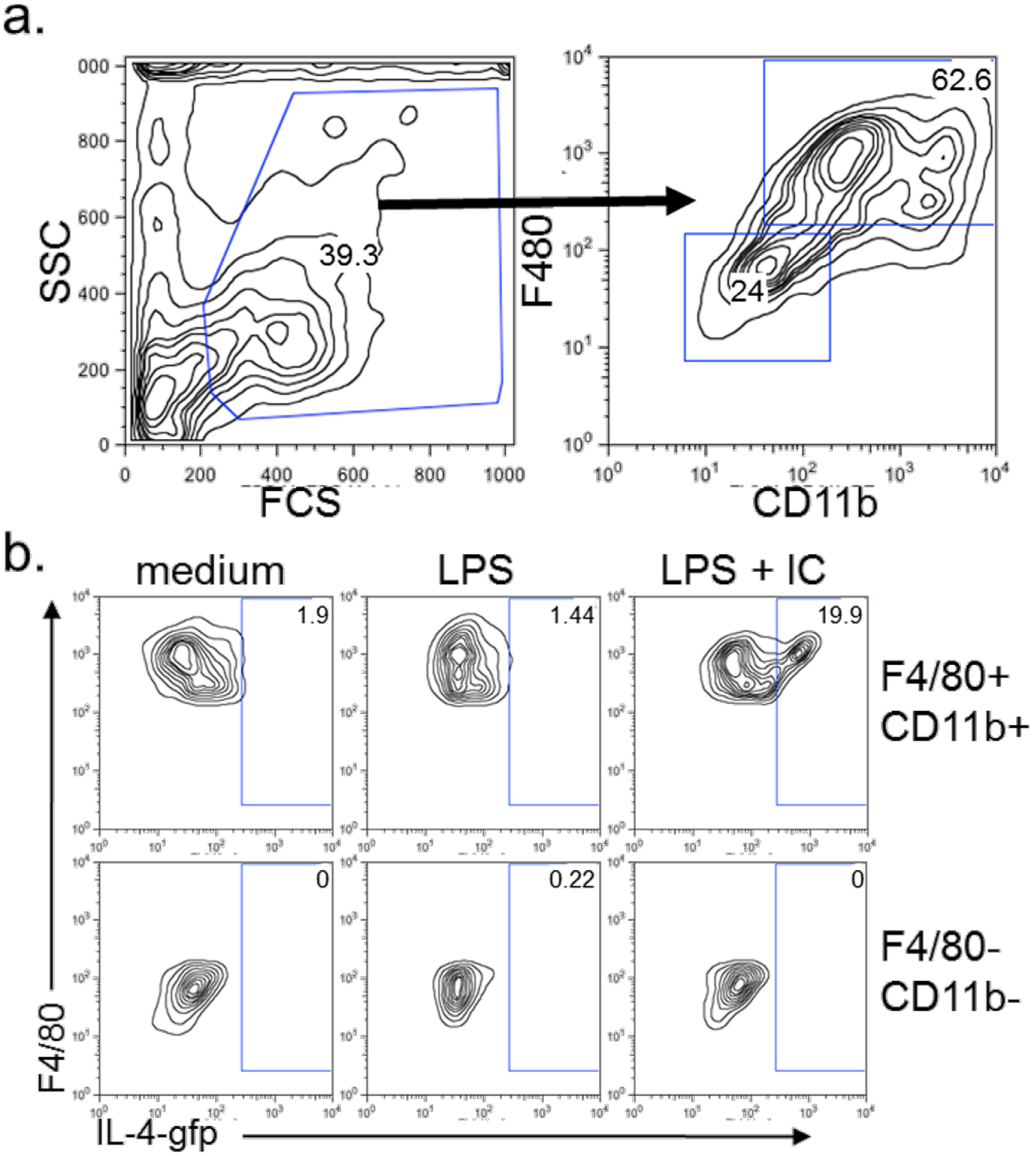
F4/80+/CD11b+ but not F4/80-CD11b- cells produce IL-4. IFN-γ-primed BMMφ (10^5^/well) from heterozygous G4 mice were stimulated with LPS (10 ng/ml) in the presence or absence of opsonized SRBC (IC) for 24 hours. (a) Live cells were selected by FSC vs SSC and this subpopulation divided into F4/80+CD11b+ and F4/80-CD11b- populations. (b) F4/80+CD11b+ cells and not F4/80-CD11b- cells produce IL-4 after stimulation with LPS + IC. Shown are representative data from 1 of 3 experiments.

To verify that the IL-4 was a product of macrophages and not the presence of another cell type that remained after isolation, we assessed IL-4 production by flow cytometry using BMMφ from mice, which produce GFP under the IL-4 promoter (i.e. G4 mice) [11]. Using these mice, it was found that type II (LPS + IC) but not classical (LPS) activation induced IL-4 production and that the cytokine was produced by cells co-expressing CD11b and F4/80 (Figure 2b and c and Figure 3). Additionally, when cells from these cultures were examined by immunofluorescent microscopy, the IL-4-producing GFP^+^ cells were clearly mononuclear and displayed macrophage-like morphology (Figure 2d). Finally, using the IL-4-GFP mice, we observed that opsonized SRBC (immune complexes; IC) but not unopsonized SBRC (S) appeared to stimulate similar levels of IL-4 production as type II activation (LPS + IC), suggesting that the enhanced IL-4 production was not solely due to type II activation but may result from FcγR ligation. Interestingly, this enhanced IL-4 production by IC stimulation alone was not evident in our culture supernatants (Figure 2c). One possible explanation is that GFP production reaches a maximum threshold above which difference in expression levels cannot be distinguished. Alternatively, because the levels in the supernatant can be regulated by receptor binding, the difference between the two assays may reflect alteration in production versus uptake.

**Figure 4:**
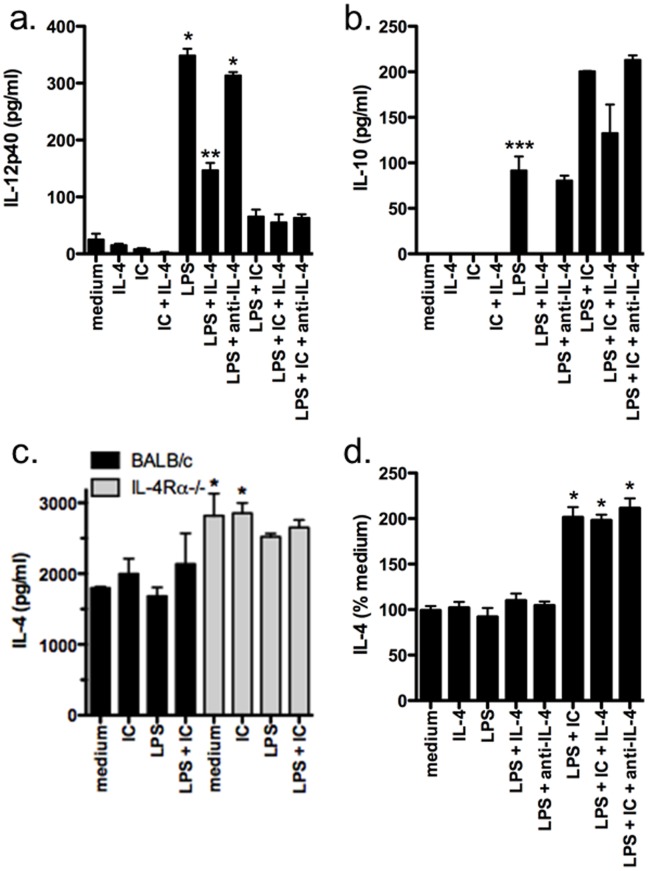
Addition of IL-4 reduces IL-12p40 and IL-10 production by BMMφ whereas neutralization of IL-4 does not alter cytokine production. (**a–c**) IFN-γ-primed BMMφ (10^5^/well) from WT (a–c) and IL-4Rα−/− (c) mice were stimulated with LPS (10 ng/ml) in the presence or absence of opsonized SRBC (IC), IL-4 (3 ng/ml), or neutralizing anti-IL-4 mAb (1 µg/ml) for 24 hours. In (c) only cultures with exogenous IL-4 (3 ng/ml) are shown. Cytokine production was measured in culture supernatants by ELISA. Shown are the means and SEM of triplicate wells from 1 of 3 representative experiments. *p<0.001 LPS or LPS + anti-IL-4 versus all other groups, **p<0.001 LPS + IL-4 versus all other groups, and ***p<0.01 LPS versus LPS + IL-4 or LPS + IC by one-way ANOVA with Bonferroni’s post-test. For (c) *p<0.05 compared to medium by Dunnett’s Multiple Comparison Test. (**d**) IFN-γ-primed BMMφ (10^5^/well) from heterozygous G4 mice were stimulated as above (a–c) and IL-4 production assessed by flow cytometry. % medium  =  (geoMFI of sample/geoMFI of medium)×100. Shown are the means and SEM of triplicate wells from 3 experiments. *p<0.01 compared to medium by Dunnett’s Multiple Comparison Test.

To determine if the macrophages were responding to the IL-4 in an autocrine fashion, CD124 (IL-4Rα) expression was assessed on classically and type II-activated BMMφ by flow cytometry. The levels of CD124 expression on BMMφ were detectable after type II activation but the levels were extremely low (Figure S3). However, when exogenous IL-4 (3 ng/ml) was added to the cultures, the levels of IL-4 detected after 8 hours were significantly reduced in BMMφ cultures from WT but not IL-4Ra-deficient mice indicating that if produced, IL-4 can be taken up by these macrophages (Figure 4c). Overall, these data suggest that the IL-4 produced by type II or IC-stimulated macrophages may signal in an autocrine fashion and contribute to regulating downstream macrophage effector functions.

### Type II Activation of Macrophages is Independent of IL-4

Given that IL-4 is produced and taken up by its signaling receptor (IL-4Rα), we assessed how IL-4 could contribute to the type II activation phenotype and cytokine production. To this end we investigated whether the addition of high levels of IL-4 or addition of a neutralizing IL-4 antibody would alter the cytokine profile of classical or type II-activated BMMφ. In particular, we were interested in determining if the neutralization of IL-4 *in vitro* would reproduce our results using IL-4Rα-deficient BMMφ. As shown in Figures 4a and b, the addition of IL-4 to BMMφ cultures stimulated with LPS (i.e. classical activation) resulted in a reduction in both IL-12p40 and IL-10 production and is consistent with previous studies [12]. However, the neutralization of IL-4 did not enhance the production of these cytokines (Figures 4a and b) as was expected given the enhanced production of IL-12p40 and IL-10 in IL-4Rα-deficient BMMφ (Figure 1a–d). Moreover, similar results were found using type II-activated BMMφ where additional IL-4 could reduce cytokine production while neutralization of IL-4 *in vitro* had no effect (Figure 4a and b). Finally, because type II activation also leads to a distinct alteration in the expression of several co-stimulatory and activation markers on macrophages (i.e. reduction in CD40 and PD-L1) [3], we investigated how IL-4 regulated the expression of CD40 and PD-L1 on classical and type II-activated BMMφ. We found that the addition or neutralization of IL-4 did not alter CD40 or PD-L1 expression and that WT and IL-4Rα-deficient BMMφ had similar levels of CD40 and PD-L1 after type II or classical activation, respectively (Figure S4 and data not shown). As expected, PD-L2 expression was significantly induced by the addition of IL-4 [13]; however, neither classical nor type II-activated BMMφ expressed PD-L2 even in the presence of exogenous IL-4 (Figure S4). Taken together these data suggest that the type II activation phenotype is independent of IL-4.

To determine if exogenous IL-4 could enhance the production of IL-4 by macrophages, we assessed whether the addition of IL-4 or a neutralizing IL-4 antibody altered the expression of IL-4 in IL-4-GFP BMMφ. As shown in Figure 4d, the addition of IL-4 did not enhance IL-4 production by type II or classically activated BMMφ. Furthermore, during the period of culture, IL-4 did not promote a significant upregulation of CD124 under any condition (data not shown). Similar results were found when IL-4 was neutralized *in vitro* (Figure 4d). These results suggest that while high levels of IL-4 may alter cytokine production by macrophages, the low levels produced by type II-activated macrophages are not responsible for the cytokine profile. Moreover, the differences in IL-12 and IL-10 production by BMMφ from WT and IL-4Rα-deficient mice are not due to autocrine IL-4 production during the *in vitro* culture. The minimal effect of IL-4 on the macrophages may be due to the extremely low levels of CD124 expressed. In contrast, other cells (e.g. T cells) that express higher levels of CD124 may be more responsive to the low levels of lL-4 produced by type II activated macrophages.

### IL-4 Produced during Derivation of BMMφ may Result in Reduced Cytokine Responses by WT Compared to IL-4Rα-deficient BMMφ

**Figure 5:**
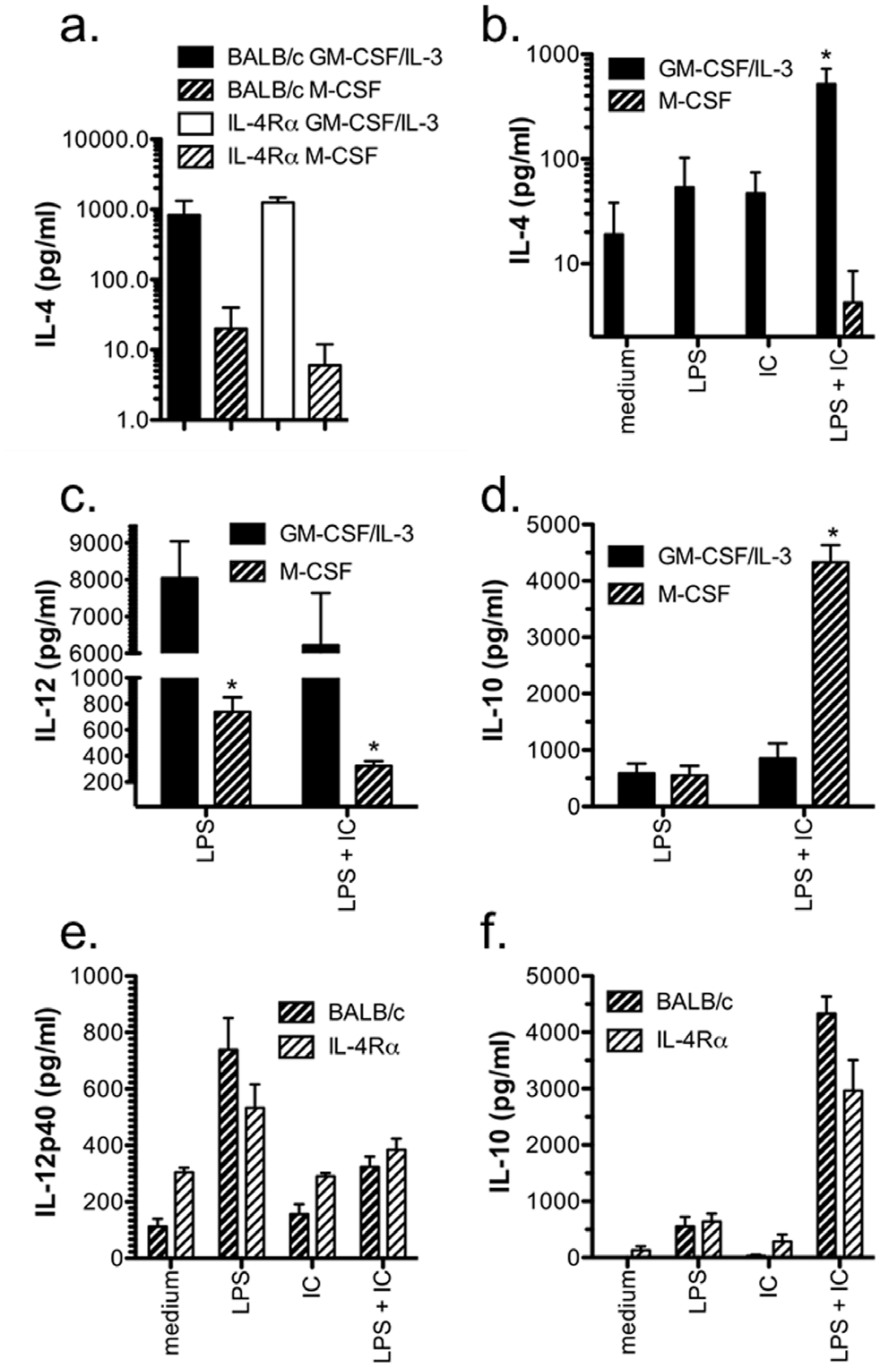
GM-CSF plus IL-3-derived BMMφ differentiate in a more IL-4 rich environment and produce higher IL-12p40 and IL-4 levels upon stimulation compared to M-CSF-derived BMMφ. GM-CSF plus IL-3 or M-CSF-derived BMMφ (10^5^/well) from WT (a–f) and IL-4Rα−/− (a, e, and f) mice were primed overnight with IFN-γ and stimulated with LPS (10 ng/ml) in the presence or absence of opsonized SRBC (IC) for 24 hours. (**a**) IL-4 in the supernatants of the derivation cultures after harvesting of the mature macrophages was assessed by ELISA. p<0.05 by repeated measures ANOVA. (**b–d**) IL-4, IL-12, and IL-10 levels were assessed in the culture supernatants of GM-CSF plus IL-3 or M-CSF –derived WT macrophages by ELISA. *p<0.01 compared to all other groups (b) or GM-CSF/IL-3 compared to M-CSF (c and d) by one way ANOVA with Bonferroni’s multiple comparison test. (**e and f**) M-CSF-derived macrophages from WT and IL-4Rα-deficient mice produce similar levels of IL-12 and IL-10 as assessed by ELISA. (**a–f**) Shown are the means and SEM of 3 independent experiments.

Our finding that the production of IL-4 by BMMφ was not responsible for the difference in cytokine production by WT and IL-4Rα-deficient BMMφ suggests that during early haematopoetic differentiation of macrophages, signals through IL-4Rα may be important in determining the quantity and range of cytokines produced after full differentiation into mature BMMφ. This potential pathway is supported by a recent study by Kuroda *et al* who found that during the derivation of macrophages from bone marrow progenitors with GM-CSF or IL-3, IL-4 produced by basophils could drive an alternative macrophage activation state [14]. To test if the derivation conditions we used (GM-CSF plus IL-3) promoted IL-4 production, we compared IL-4 levels in the supernatants of the bone marrow cultures derived with GM-CSF plus IL-3 to those with M-CSF. In supernatants from both WT and IL-4Rα-deficient cultures, significantly more IL-4 was produced when GM-CSF plus IL-3 was used than M-CSF (Figure 5a). Moreover, BMMφ derived using GM-CSF plus IL-3 had a greater propensity to produce IL-4 after type II activation than M-CSF-derived macrophages, which produced only minimal levels of IL-4 (Figure 5b). As expected, both derivation methods produced BMMφ that could be type II activated; however, GM-CSF plus IL-3-derived macrophages produced significantly more IL-12p40 upon classical activation while M-CSF-derived macrophages produced significantly higher IL-10 upon type II activation (Figure 5c and d). Finally, when comparing M-CSF-derived macrophages from WT and IL-4Rα-deficient mice, there was no clear difference in IL-12 and IL-10 production as seen with GM-CSF plus IL-3-derived macrophages (Figure 5e and f) suggesting that the high levels of IL-4 produced during the differentiation with GM-CSF plus IL-3 may be responsible for the observed reduction in IL-12p40 and IL-10 production after classical or type II activation of WT compared to IL-4Rα-deficient BMMφ.

### Other Type II-activating Stimuli Induce IL-4 Production by Macrophages

**Figure 6:**
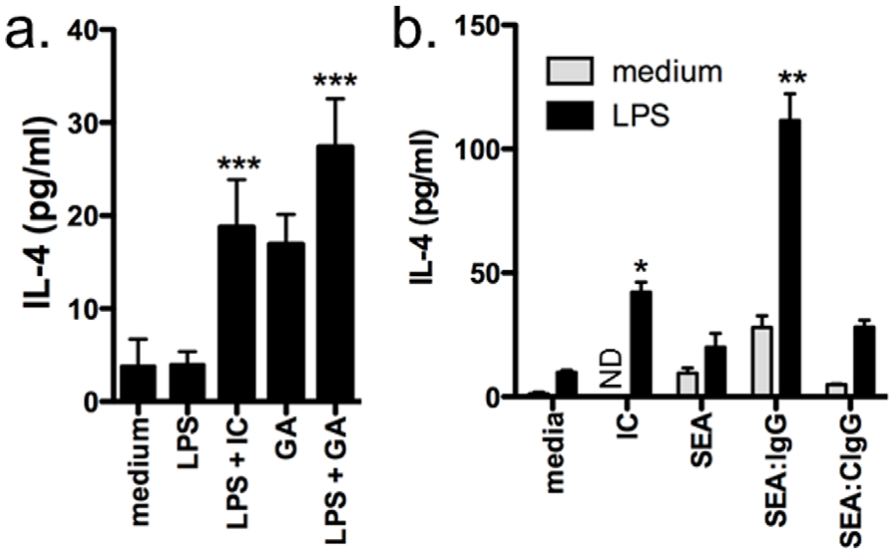
Other type-II activating products stimulate IL-4 production by macrophages. IFN-γ-primed BMMφ (10^5^/well) from WT BALB/c (a) or C57BL/6 (b) mice were stimulated with LPS (10 ng/ml) in the presence or absence of opsonized SRBC (IC), glatiramer acetate (200 ng/ml), SEA (5 µg/ml) or SEA opsonized with IgG purified from infected mice (SEA:IgG) or control IgG (SEA:CIgG; 5 µg/ml: 20 µg/ml) for 24 hours. Cytokine production was measured in culture supernatants by ELISA. Shown are the means and SEM of triplicate wells from 1 of 2 representative experiments. *p<0.05 compared to LPS alone and **p<0.001 compared to al other groups by one-way ANOVA with Bonferroni’s post test. ***p<0.05 compared to medium by Dunnett’s Multiple Comparison Test.

Type II activation is commonly induced using opsonized SRBC [3], but other IC and compounds have also been reported to induce type II activation including opsonized ovalbumin [7], opsonized schistosome egg antigens (SEA) from *Schistosoma mansoni* [15], and glatiramer acetate (a common treatment for multiple sclerosis) [16], [17]. To determine whether the production of IL-4 by type II-activated macrophages was specific to the use of opsonized SRBC, we measured IL-4 production by BMMφ type II-activated with glatiramer acetate and LPS. In comparison to BMMφ activated with opsonized SRBC and LPS, type II activation by glatiramer acetate induced a similar level of IL-4 production (Figure 6a). Interestingly, glatiramer acetate alone resulted in a similar level of IL-4 as glatiramer acetate and LPS (Figure 6a). In contrast, while glatiramer acetate and LPS reduced IL-12p40 production and increased IL-10, glatiramer acetate alone did not induce BMMφ to produce either IL-12p40 or IL-10 (Figure S5). Glatiramer acetate is known to enhance Th2 responses in EAE, the animal model of MS [18]; however, the mechanism by which this occurs in unknown. Our results suggest that glatiramer acetate drives type II activation of macrophages and possibly microglia and thus induces the innate production of IL-4. This IL-4 in turn may promote immune deviation and Th2 response development. Recent work has confirmed that microglia produce IL-4 in the CNS during EAE [19] and together with our results, these findings suggest that type II activation by glatiramer may further promote IL-4 production by microglia in the CNS.

Given the known Th2-promoting ability of schistosome eggs [20], we assessed if opsonized SEA upregulated IL-4 production by C57BL/6 macrophages. In a manner similar to BALB/c macrophages, type II-activated macrophages from C57BL/6 mice produced significant but modest levels of IL-4 (Figure 6b), and exposure to opsonized SEA (SEA:IgG) greatly enhanced IL-4 production. SEA alone induced a small increase in IL-4, which was further enhanced by LPS, but these levels were significantly less than those induced by opsonized SEA. Furthermore, no increase in IL-4 production was found when control IgG was incubated with SEA (SEA:CIgG) indicating that the effect is dependent on IC (Figure 6b). Finally, as observed with glatiramer acetate and opsonized SRBC, the opsonized SEA alone induced a modest but significant level of IL-4 (Figure 6b). Together these findings suggest that opsonized SEA may be involved in the Th2-promoting ability of *S. mansoni* by promoting type II activation and IL-4 production by macrophages.

### Conclusions

Overall, our results are the first to indicate that type II activation induces production of low but significant levels of IL-4 by macrophages and that this production is common to all type II-inducing agents tested. Furthermore, we showed that the classical and type II activation profile is independent of IL-4 and neutralization of IL-4 did not enhance IL-10 or IL-12p40 cytokine production to the level of the IL-4Rα-deficient BMMφ. Instead the difference in the capacity of BMMφ to produce these cytokines may be determined during their differentiation from bone marrow progenitors where the use of GM-CSF plus IL-3 leads to an IL-4 rich environment. Finally, if the Th2-promoting effects of type II activation are a result of macrophage-derived IL-4 acting upon the CD4 T cell during Th subset differentiation, a new pathway by which innate cells promote Th2 subset differentiation will have been identified and research is currently underway to confirm this idea. This work thus reveals another mechanism by which innate cells may direct and shape adaptive responses and opens up a new target for therapies designed to regulate dysfunctional immune responses.

## Materials and Methods

### Mice

WT and IL-4Rα^−/−^ BALB/c male and female mice were bred at the Victoria University of Wellington animal facility and WT C57BL/6 mice were bred at the Malaghan Institute of Medical Research animal facility in Wellington, New Zealand. GFP/IL-4 knockin (G4) mice were generously provided by Graham Le Gros (Malaghan Institute), and their generation has been previously described [11]. All animals were utilized at 8–12 wk of age. All experimental procedures used in this study were approved by the Victoria University of Wellington Animal Ethics Committee (protocol #2008R12).

### Cellular Isolation and in vitro Culture

Bone marrow-derived macrophages (BMMφ) were derived as described [21]. Briefly, progenitor cells were flushed from the femurs of mice, the adherent cells removed, and the remaining cells cultured with GM-CSF and IL-3 (5 ng/ml of each; PeproTech, Rocky Hill, NJ) or M-CSF (5 ng/ml; R & D Systems, Minneapolis, MN) for 7–10 days. The adherent cells (i.e. macrophages) were collected after incubation in cold Dulbecco’s PBS (Ca^2+^ and Mg^2+^-free; Invitrogen, Auckland, New Zealand). For *in vitro* macrophage cultures, BMMφ (10^5^/well) were cultured in complete medium containing DMEM, 10% FCS, 100 U/ml penicillin plus 100 mg/ml streptomycin, 10 mM Hepes, 2 mM L-glutamine, and 50 µM 2-ME (all from Invitrogen) in 96-well, flat-bottomed plates (BD Biosciences, Franklin Lakes, NJ). After overnight stimulation with IFN-γ (20 U/ml; BD Biosciences), BMMφ were cultured with LPS (10 ng/ml; Sigma, St. Louis, MO) in the presence or absence of type II-inducing stimuli, IL-4 (3 ng/ml), or neutralizing rat anti-IL-4 mAb clone 11B11 (BD Bioscience).

SRBC (freshly isolated from healthy sheep, Taylor Preston Ltd., Wellington, New Zealand) were opsonised with rabbit anti-SRBC IgG polyclonal Ab (Sigma). Briefly, SRBC were incubated with anti-SRBC antibody at a non-agglutinating concentration for 30 minutes at 37°C. Immune complexes (IC) were washed of free antibody and added to BMMφ at a 10∶1 ratio (IC to BMMφ). Soluble egg antigen (SEA) was prepared as previously described [22] and allowed to complex for 30 minutes with IgG purified from *Schistosoma mansoni*-infected mice using a Protein G column (Sigma). Glatiramer acetate was kindly donated by B. Thomas Bäckström (while at Malaghan Institute of Medical Research, Wellington, New Zealand).

### Fluorescence Microscopy

After 24 hour stimulation with classical or type II-activating stimuli, G4 BMMφ were spun onto slides using a cytospin (Thermo Scientific Shandon, Waltham, MA) mounted with DAPI containing ProLong Gold anti-fade reagent (Invitrogen), and viewed using a fluorescent microscope.

### Cytokine Assays

IL-12p40, IL-10, IL-13, and IL-4 levels in culture supernatants were assessed by sandwich ELISA. For IL-12p40, IL-10, and IL-4, matched antibody pairs and recombinant standards were purchased from BD Bioscience and used according to the manufacturer’s recommendations. For IL-13, the IL-13 DuoSet ELISA kit (R&D Systems) was used according to the manufacturer’s protocol.

### Flow Cytometry

After *in vitro* activation, surface marker expression on BMMφ was assessed by flow cytometry. After incubation with Fc Block (BD Bioscience), cells were treated with rat anti-F4/80 (Serotec, Oxford, UK), anti-CD11b (BD Biosciences), rat anti-CD40 (BD Biosciences), rat anti-PD-L1 (eBioscience, San Diego, CA), rat anti-PD-L2 (eBioscience), or anti-CD124 (BD Biosciences). Isotype control rat antibodies (BD Biosciences) were used to determine background fluorescence. The samples were analyzed using a FACScan flow cytometer and CELL Quest software (Becton Dickinson).

### Statistical Analyses

Data were analyzed using one-way ANOVA with Bonferroni’s post-test or Dunnett’s post-test (to compare to a control condition). P values <0.05 were considered significant.

## Supporting Information

Figure S1 **IL-4 is produced by type II activated macrophages 8 and 24 hours post activation.** BMMφ (10^5^/well) from WT C57BL/6 mice were stimulated with LPS (10 ng/ml) in the presence or absence of opsonized SRBC (IC) for 8 or 24 hours. Cytokine production was measured in culture supernatants by IL-4 ELISA. Shown are the means and SEM of triplicate wells.
(TIF)

Figure S2 **IL-13 is not induced after classical or type II activation of macrophages.** BMMφ (10^5^/well) from WT BALB/c mice were stimulated with LPS (10 ng/ml) in the presence or absence of opsonized SRBC (IC) or IL-4 (3 ng/ml) for 24 hours. Cytokine production was measured in culture supernatants by IL-13 ELISA. Shown are the means and SEM of triplicate wells.
(TIF)

Figure S3 **CD124 expression is detectable but extremely low on IC and LPS + IC-treated BMMφ.** BMMφ (10^5^/well) from WT BALB/c mice were stimulated with LPS (10 ng/ml) in the presence or absence of opsonized SRBC (IC) for 24 hours. Live cells were selected by FSC vs SSC. Shown is the CD124 expression on the F4/80+CD11b+ population. The geometric mean fluorescent intensity for each sample is indicated in the box.
(TIF)

Figure S4 **Type II activated macrophages do not express PDL-2 and have reduced expression of PDL-1.** BMMφ (10^5^/well) from WT and IL-4Rα-deficient mice were stimulated with LPS (10 ng/ml) in the presence or absence of opsonized SRBC (IC) or IL-4 (3 ng/ml) for 24 hours. Live cells were selected by FSC vs SSC. Shown are the PDL-2 and PDL-1 expression on the F4/80+CD11b+ population. The geometric mean fluorescent intensity for each sample is indicated in the box.
(TIF)

Figure S5 **GA-generated type II macrophages show a modest reduction in IL-12 and modest increase in IL-10 compared to IC-generated type II macrophages.** BMMφ (10^5^/well) from WT BALB/c mice were stimulated with LPS (10 ng/ml) in the presence or absence of opsonized SRBC (IC) or GA (100 µg/ml) for 24 hours. Cytokine production was measured in culture supernatants by IL-12p40 and IL-10 ELISA. Shown are the means and SEM of triplicate wells.
(TIF)
