# Supplementary figures and images for: Type II-Activated Murine Macrophages Produce IL-4
Source: PLoS One. 2012 Oct 5;7(10):e46989. doi: 10.1371/journal.pone.0046989 (PMC3465319; doi:10.1371/journal.pone.0046989)

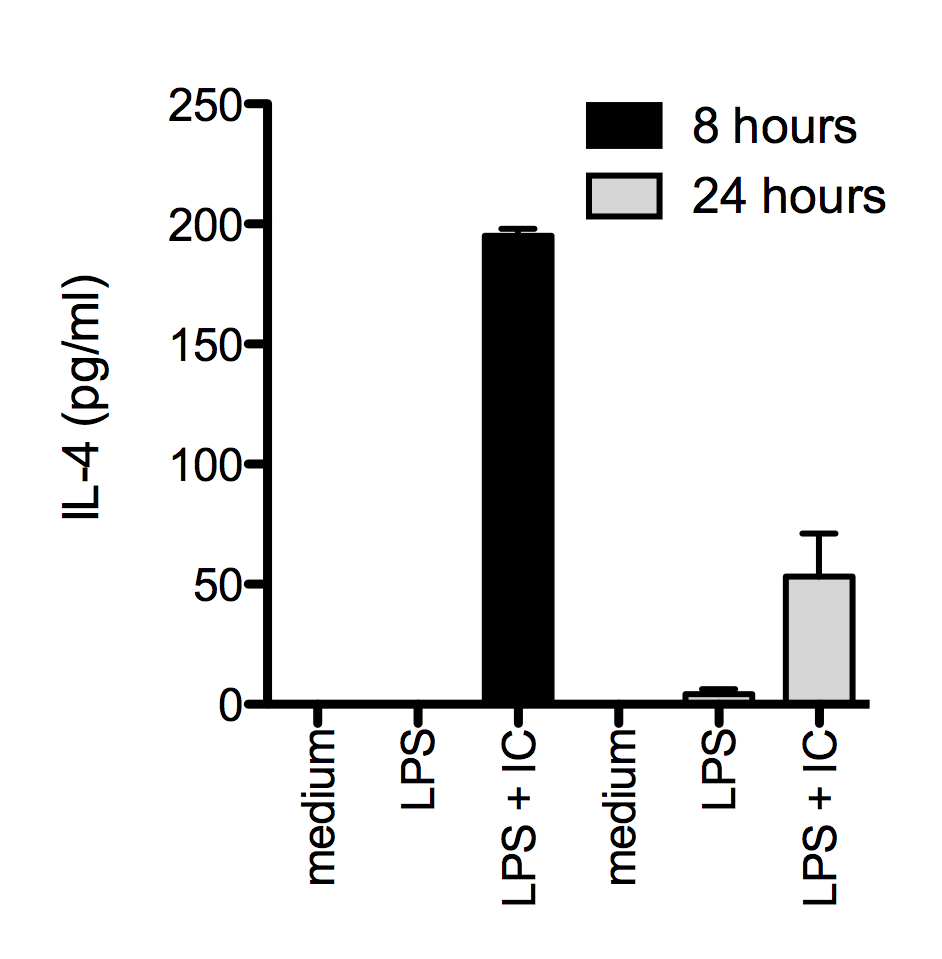

Supplement: Figure S1 — IL-4 is produced by type II activated macrophages 8 and 24 hours post activation. BMMφ (105/well) from WT C57BL/6 mice were stimulated with LPS (10 ng/ml) in the presence or absence of opsonized SRBC (IC) for 8 or 24 hours. Cytokine production was measured in culture supernatants by IL-4 ELISA. Shown are the means and SEM of triplicate wells. (TIF) [file pone.0046989.s001.tif]

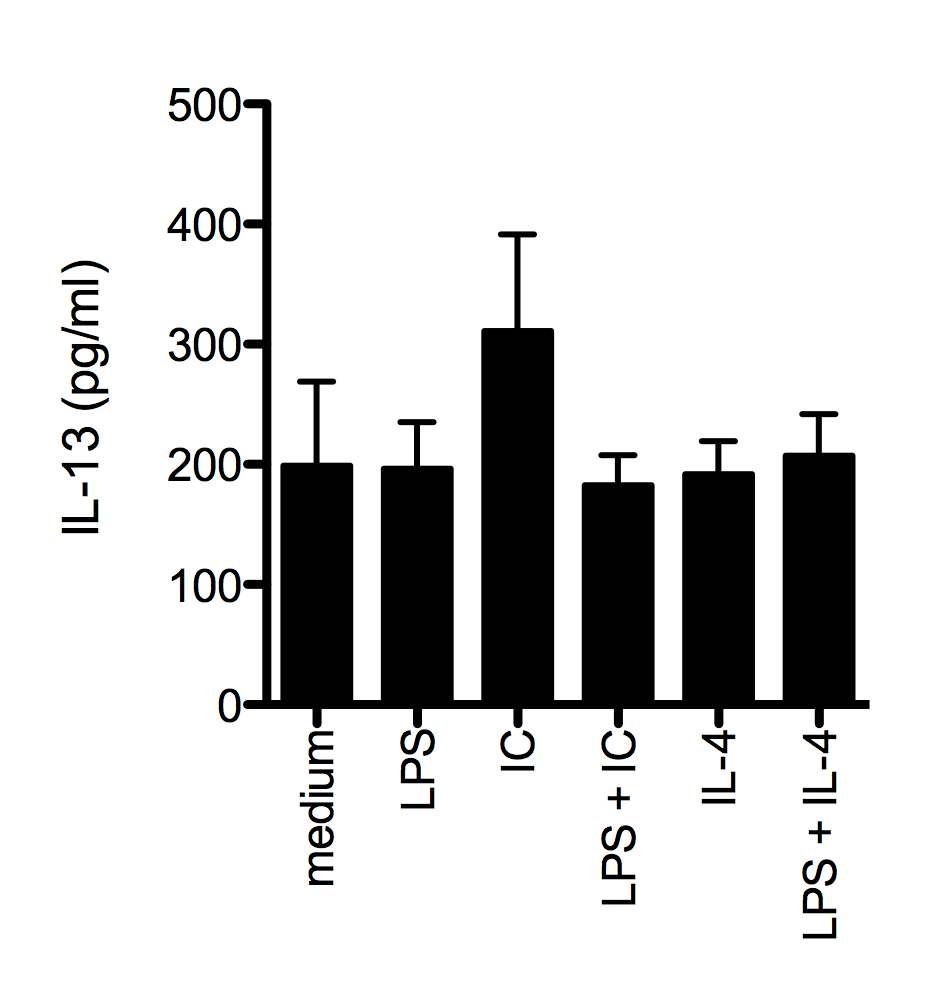

Supplement: Figure S2 — IL-13 is not induced after classical or type II activation of macrophages. BMMφ (105/well) from WT BALB/c mice were stimulated with LPS (10 ng/ml) in the presence or absence of opsonized SRBC (IC) or IL-4 (3 ng/ml) for 24 hours. Cytokine production was measured in culture supernatants by IL-13 ELISA. Shown are the means and SEM of triplicate wells. (TIF) [file pone.0046989.s002.tif]

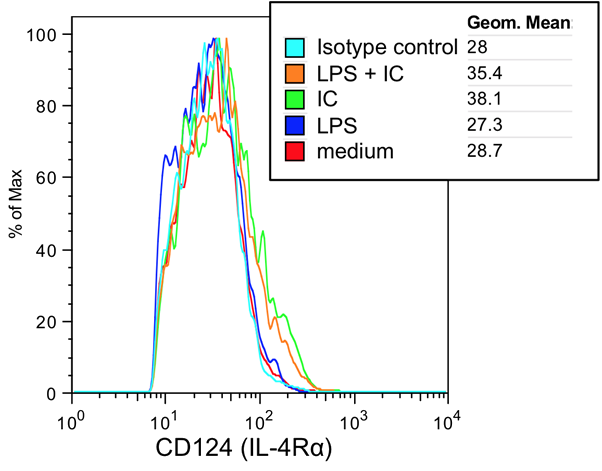

Supplement: Figure S3 — CD124 expression is detectable but extremely low on IC and LPS + IC-treated BMMφ. BMMφ (105/well) from WT BALB/c mice were stimulated with LPS (10 ng/ml) in the presence or absence of opsonized SRBC (IC) for 24 hours. Live cells were selected by FSC vs SSC. Shown is the CD124 expression on the F4/80+CD11b+ population. The geometric mean fluorescent intensity for each sample is indicated in the box. (TIF) [file pone.0046989.s003.tif]

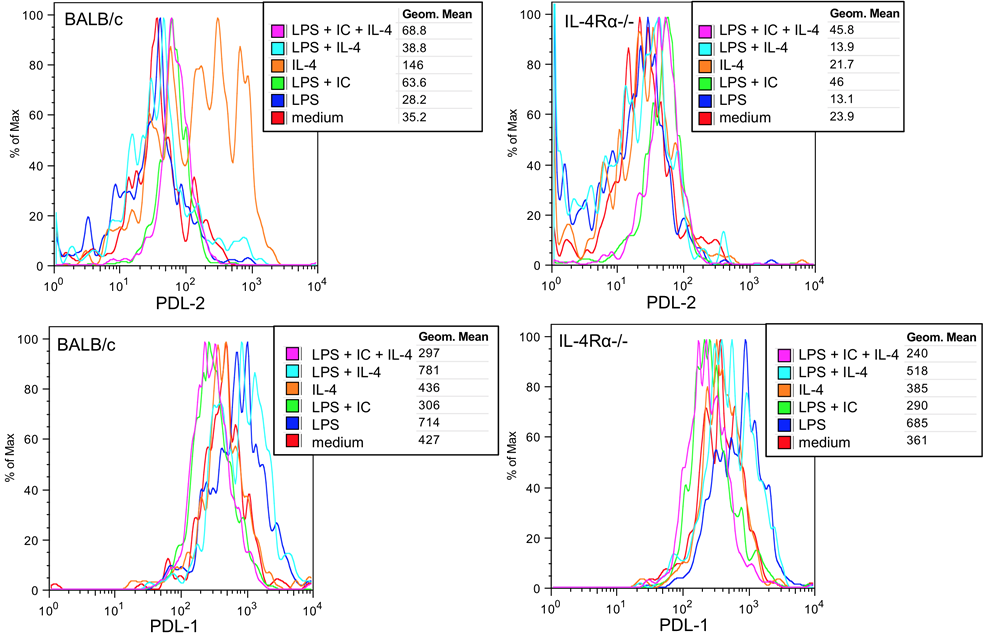

Supplement: Figure S4 — Type II activated macrophages do not express PDL-2 and have reduced expression of PDL-1. BMMφ (105/well) from WT and IL-4Rα-deficient mice were stimulated with LPS (10 ng/ml) in the presence or absence of opsonized SRBC (IC) or IL-4 (3 ng/ml) for 24 hours. Live cells were selected by FSC vs SSC. Shown are the PDL-2 and PDL-1 expression on the F4/80+CD11b+ population. The geometric mean fluorescent intensity for each sample is indicated in the box. (TIF) [file pone.0046989.s004.tif]

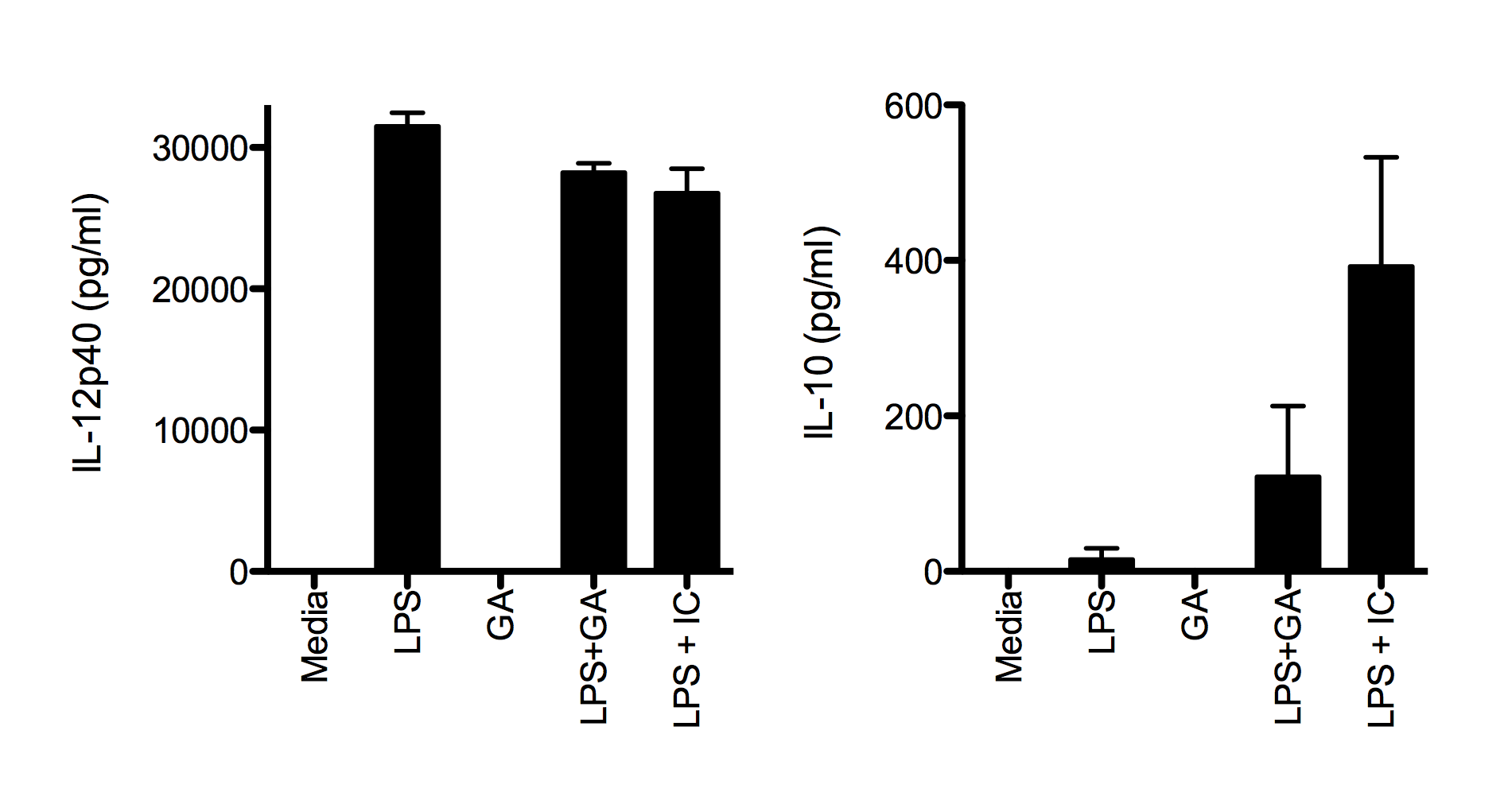

Supplement: Figure S5 — GA-generated type II macrophages show a modest reduction in IL-12 and modest increase in IL-10 compared to IC-generated type II macrophages. BMMφ (105/well) from WT BALB/c mice were stimulated with LPS (10 ng/ml) in the presence or absence of opsonized SRBC (IC) or GA (100 µg/ml) for 24 hours. Cytokine production was measured in culture supernatants by IL-12p40 and IL-10 ELISA. Shown are the means and SEM of triplicate wells. (TIF) [file pone.0046989.s005.tif]
